# Antimicrobial Stewardship from Health Professionals’ Perspective: Awareness, Barriers, and Level of Implementation of the Program

**DOI:** 10.3390/antibiotics11010099

**Published:** 2022-01-14

**Authors:** Haya Nassar, Rana Abu-Farha, Muna Barakat, Eman Alefishat

**Affiliations:** 1 Department of Clinical Pharmacy and Therapeutics, Faculty of Pharmacy, Applied Science Private University, Amman 11931, Jordan; hayanassar32@gmail.com (H.N.); r_abufarha@asu.edu.jo (R.A.-F.); m_barakat@asu.edu.jo (M.B.); 2 Department of Pharmacology, College of Medicine and Health Science, Khalifa University of Science and Technology, Abu Dhabi P.O. Box 127788, United Arab Emirates; 3 Center for Biotechnology, Khalifa University of Science and Technology, Abu Dhabi P.O. Box 127788, United Arab Emirates; 4 Department Biopharmaceutics and Clinical Pharmacy, Faculty of Pharmacy, The University of Jordan, Amman 11942, Jordan

**Keywords:** antimicrobial stewardship, perception, practice, tertiary hospitals, Jordan

## Abstract

This study aimed to evaluate health professionals’ perceptions regarding the level of implementation of the Antimicrobials Stewardship (AMS) programs in Jordanian tertiary hospitals and to assess the perceived barriers to its implementation. During this cross-sectional study, a total of 157 healthcare providers agreed to participate (response rate 96.3%). Participants were asked to complete an electronic survey after meeting them at their working sites. Only 43.9% of the healthcare providers (*n* = 69) reported having an AMS committee in their hospital settings. The results suggested that private hospitals have significantly better AMS implementation compared to public hospitals among four areas (*p* ≤ 0.05). Moreover, the results showed that the most widely available strategies to implement AMS were infectious disease/microbiology advice (*n* = 112, 71.3%), and treatment guidelines (*n* = 111, 70.7%). Additionally, the study revealed that the main barrier to AMS implementation was the lack of information technology support (*n* = 125, 79.6%). These findings could draw managers’ attention to the importance of AMS and support the health care provider’s practice of AMS in Jordanian tertiary hospitals by making the right decisions and the required modifications regarding the strategies needed for the implementation of AMS programs.

## 1. Introduction

Antimicrobial agents have saved millions of lives and controlled the majority of infectious diseases that swept the world [1]. Their antimicrobial activity is essential in the fight against infectious agents [2]. However, microorganisms readily adapt to their environments and can develop ways to survive and grow in the presence of antimicrobial agents [3]. This can lead to the development of antimicrobial resistance, which is considered a great threat to health systems worldwide [4,5].

Every year more than two million individuals become infected with resistant microorganisms in the United States, according to the Centers for Disease Control and Prevention (CDC) [5]. In Europe, antimicrobial resistance contributes to approximately 25,000 deaths yearly [6]. Furthermore, a recent review showed that the additional cost of antimicrobial resistance could reach £20,000 per patient in hospital settings [7]. Accordingly, the world is experiencing a real crisis and significant challenges since the spreading of antimicrobial resistance leads to a decrease in the efficacy of antibiotics that play a significant role in saving human life [8,9]. Thus, the World Health Organization (WHO) has long propagated the need for the coordinated global potential to control resistance to antimicrobials [5,10]. Therefore, international health care organizations and health agencies have recommended developing effective tools or programs to manage the development of antimicrobial resistance [11]. Antimicrobial stewardship (AMS) programs are the most effective programs that aim to optimize patient safety, quality of care and minimize antimicrobial resistance [12]. In addition, they significantly contribute to the healthcare system through promoting and monitoring antimicrobial agents [13]. The implementation of such programs is usually achieved via a multidisciplinary antimicrobial team consisting of physicians, pharmacists, microbiologists, epidemiologists, and infectious disease specialists with sufficient experience in their respective fields [14]. The roles of physicians in AMS mainly include the the prescription of antimicrobials and the overall supervision of the AMS process [15], while pharmacists’ main role is reviewing the medication charts to evaluate the indications, doses, and duration of the prescribed antimicrobials and to monitor any possible allergies or side effects [16]. Furthermore, nurses ensure the appropriate administration of antimicrobials and educate the patient [17].

Accordingly, health care providers should have an adequate knowledge, awareness, acceptability, and understanding about AMS to implement the program successfully in their institutions. A recent study by Kpokiri et al. (2022) in Ghana demonstrated that health care providers have good knowledge about the AMS program and that continuous training sessions have a significant impact on their understanding, practice, and AMS skills at the tertiary hospitals [18]. The same study mentioned barriers that could interfere with the program’s implementation, such as ‘Lack of funding in healthcare,’ ‘Staff shortages,’ and ‘Failure to enforce laws’. Several measures have been reported to overcome barriers to AMS implementation, including education empowerment, continuous professional training, antibiotics use audits, and increased staff members and workforce [18].

In the context of Jordan, the Jordanian Ministry of Health has released a national action plan (NAP) for the following years (2018–2022) to combat antimicrobial resistance [19]. This NAP relates to all the objectives set up in WHO global action plan. One goal the NAP aimed to achieve within the five-year plan is enhancing the proper use of antimicrobial agents in health, animals, and food. However, despite all of those efforts, there is an apparent problem in healthcare providers’ awareness and perception of these rules and procedures. In addition, implementing these programs in hospitals has not been achieved [19].

The best way to achieve successful AMS implementation is to have an adequate organizational structure, commitment, and resources availability [10]. In addition, the sufficient awareness of healthcare providers of AMS principles and their accountability for program administration and outcomes is also necessary to ensure efficient implementation of AMS [13]. Thus, the main goal of this study is to investigate the era of AMS from health professionals’ perspective at the Jordanian tertiary hospitals (private and public), including the awareness, perceived barriers, the availability, and the level of implementation of the AMS program.

## 2. Methods

### 2.1. Study Design and Participants

This cross-sectional survey study was conducted to assess and investigate health professionals’ perceptions at different Jordanian tertiary hospitals regarding the level of implementation of the AMS program, their awareness, and the perceived barriers to its implementation. During the study period, of November 2021, a convenient sample of healthcare providers, including physicians, pharmacists, and nurses working at different Jordanian hospitals, was recruited to participate in this study. Healthcare providers were recruited if working in tertiary hospitals where specialized care is provided.

The questionnaire was uploaded electronically via the google form platform, and was distributed electronically to the participants after meeting them at their working sites. Willing participants could open a link to initially view the study aim, the potential benefits of the survey, the confidentiality of data, and the voluntariness of participation. After that, they were asked to provide their electronic consent before proceeding to the study.

### 2.2. Questionnaire Development and Data Collection

The study questionnaire was developed based on the CDC Core Elements of the Hospital Antibiotic Stewardship Programs [20]. The CDC questionnaire was condensed and revised to include feasible elements in the Jordanian setting. The questionnaire was designed in the English language. The initial draft was face and content validated by a group of four academics to evaluate the relevance of questions to the Jordanian setting and the comprehensibility and clarity of the items included. Then, one of the researchers (R. A.) incorporated the comments received by the academics to develop the final version of the questionnaire. The final questionnaire (File S1) was divided into three primary domains, which include (1) The socio-demographic information, which included age, gender, experience, and discipline, and information about the hospital classification and location was included in this section. (2) The second section included questions to evaluate healthcare providers’ perceptions of the level of implementation of some of the core elements of hospitals’ AMS programs, including organizational structure, resources, reporting, and education. (3) The last section evaluated the healthcare providers’ perception of AMS’s importance and the barriers preventing them from delivering an AMS.

Finally, the internal reliability was tested using Cronbach’s alpha measure, which yielded 0.872, indicating that the scale has an acceptable internal consistency.

### 2.3. Ethical Consideration

The study protocol was approved by the Institutional Review Board (IRB) committee at the Faculty of Pharmacy at Applied Science Private University (2021-PHA-38). The study was conducted following the ethical standards outlined in the World Medical Association Declaration of Helsinki guideline [21]. Electronic consents were obtained from all healthcare providers who agreed to participate in the study. Additionally, participants were informed about the study objectives, confidentiality of responses, and their right to evacuate from the study.

### 2.4. Statistical Analysis

Data were entered and analyzed using the Statistical Package for the Social Sciences (SPSS), Version 25.0 (SPSS Inc., Chicago, IL, USA). The level of statistical significance was set at a *p*-value of 0.05. Descriptive statistics were used to analyze the demographic data: medians and interquartile ranges (IQR) for continuous variables, whereas categorical variables were illustrated by frequencies and percentages. The Shapiro–Wilk test tested the normality; *p*-value > 0.05 indicating a normally distributed continuous variable).

Chi-squared test was conducted to screen the difference between healthcare working in private hospitals and those working in public hospitals in their responses regarding the implementation of AMS at the hospitals they are working in. A *p* ≤ 0.05 was considered statistically significant. Cronbach’s α was used to evaluate the reliability of the questionnaire, i.e., that the scales constructed are fit for its purpose, with values ≥ 0.7 indicating acceptable internal consistency [22].

## 3. Results

During the study period, a total of 163 healthcare providers from 9 Jordanian tertiary hospitals were invited to participate in this study; among them, 157 agreed to participate (response rate 96.3%). Healthcare providers were classified as follows: 53 physicians (33.8%), 51 pharmacists (32.5%), and 53 nurses (33.8%). Participants had a median age of 31.0 years (IQR = 10.0), and females represented 59.9% of them (*n* = 94). Healthcare providers had a median experience of 5.0 years (IQR = 7.0). Around two-thirds of healthcare providers (*n* = 104, 66.2%) were working in private hospitals, and the majority were working in hospitals located in the central region of Jordan (*n* = 141, 89.8%). For more details about the socio-demographic characteristics, refer to Table 1.

**Figure 1:**
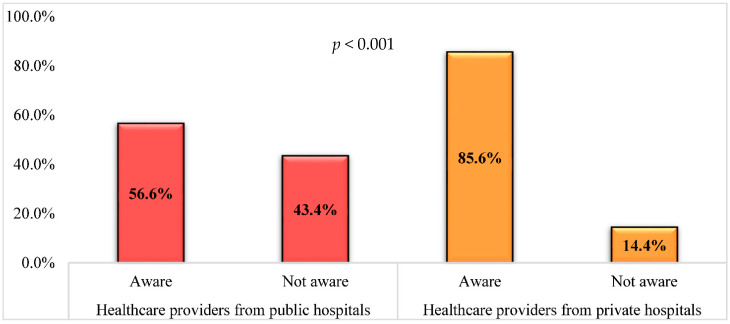
Differences between healthcare providers from public hospitals (*n* = 53) and private hospitals (*n* = 104) based on their awareness about the presence of the national action plan on antimicrobial stewardship (2018–2022) in Jordan. *p*-value was calculated using the Chi-squared test.

Healthcare providers were asked about their awareness of the presence of the NAP on AMS (2018–2022) in Jordan, where 75.8% of them (*n* = 119) reported being aware of the NAP, while the remaining 24.2% (*n* = 38) were not aware of the NAP. Healthcare providers working at the private hospitals were more aware of existing NAP at their institution compared to those working in public hospitals, as seen in Figure 1 (85.6% versus 56.6%, *p* < 0.001).

On the other hand, healthcare providers were asked about the level of implementation of AMS activities at the hospitals where they work (Table 2). Only 43.9% of the healthcare providers (*n* = 69) reported having an AMS committee in their hospital settings. Moreover, 75.2% (*n* = 118) and 62.4% (*n* = 98) of them stated that the hospitals they work in have policy and treatment guidelines to deal with antimicrobial agents, respectively.

Additionally, results depicted that more than 60.0% of the healthcare providers reported that they receive antimicrobial resistance levels/surveillance reports (*n* = 109, 69.4%), software to record antimicrobial susceptibility results (*n* = 100, 63.4%), and antimicrobial use reports (*n* = 102, 65.0%) at the hospitals they work in.

Regarding the availability of literature or evidence-based medicine about antimicrobial agents, only 60.5% of the participants (*n* = 95) reported having access to such data. A similar proportion (*n* = 95, 60.5%) also reported receiving education on optimal prescribing, adverse reactions from antibiotics, and antibiotic resistance.

When comparing the level of the implementation of AMS activities as reported by the recruited healthcare providers, results showed that private hospitals have a significantly better implementation of AMS compared to public hospitals in four areas (*p* ≤ 0.05). These areas include (1) having antimicrobial treatment guidelines, (2) using antimicrobial resistance levels/surveillance reports, (3) having access to literature or evidence-based medicine, and (4) providing education to prescribers and other relevant staff on optimal prescribing, adverse reactions from antibiotics, and antibiotic resistance.

**Figure 2:**
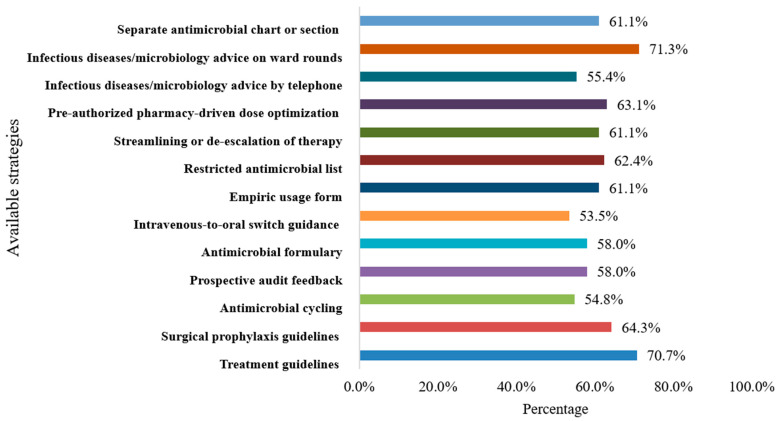
Available antimicrobial stewardship strategies at the hospitals as reported by the healthcare providers (*n* = 157).

Figure 2 illustrates the available AMS strategies at the hospitals as reported by healthcare providers. Results showed that the most available strategies were: infectious disease/microbiology advice of ward round (*n* = 112, 71.3%), treatment guidelines (*n* = 111, 70.7%), surgical prophylaxis guidelines (*n* = 101, 64.3%), and pre-authorized pharmacy-driven dose optimization (*n* = 99, 63.1%). For more details about the available strategies, refer to Figure 2.

Regarding healthcare providers’ perception towards the importance of the AMS, the majority of respondents believed that AMS would improve patient’s clinical outcomes (*n* = 134, 85.4%), would reduce antimicrobial resistance (*n* = 125, 79.6%), would improve the cost-effectiveness of health care sectors (*n* = 121, 77.1%), and would improve the collaboration between a healthcare provider (*n* = 106, 67.5%). For more details, refer to Table 3.

Finally, participants were asked to indicate their perceptions of the barriers to delivering a functional and effective AMS (Table 4). Results illustrate that the main barrier against the AMS implementation was the lack of information technology support (*n* = 125, 79.6%), followed by the lack of funding (*n* = 121, 77.1%), and the lack of sufficient healthcare providers (*n* = 119, 75.8%). The least ranked barrier was the lack of awareness of hospital administration about AMS (*n* = 103, 65.6%).

## 4. Discussion

This study investigated the era of the AMS program from health professionals’ perspective at the Jordanian tertiary hospitals (private and public), including the awareness, perceived barriers, the availability, and the level of implementation of the AMS program. In general, the study participants showed a good level of awareness but an insufficient implementation of AMS programs. The main barrier to AMS implementation was the lack of information technology support.

In Jordan, the health care system is divided into two sectors, namely, the private and public. The total number of hospitals in both sectors is 106, with 12 081 beds, and more than half are public [23]. However, our findings revealed a higher number of responses from private hospitals than public hospitals, with a significantly higher level of awareness toward the AMS program. This could be related to the increased workload in public hospitals, lack of time, motivation, and incentives with slight response tendency, as mentioned in different studies [24,25,26,27].

In parallel, our results revealed an excellent general awareness toward AMS; furthermore, private hospitals’ health care providers demonstrated a significantly higher level of awareness towards AMS programs than the public sector. Conversely, a study conducted by Baraka et al. showed that most healthcare providers reported a lack of awareness and experience of AMS programs [28]. Unfortunately, insufficient published literature compares the AMS awareness and implementation between private and public hospitals. Nevertheless, a systematic review was published in 2012 comparing the performance of private and public healthcare systems in low- and middle-income countries. This review did not support the claim of superiority of the private sectors in the efficiency, accountability, and medical effectiveness over the public sector. Still, it confirmed that the public sector frequently faces a deficiency in timeliness and hospitality towards patients [29]. Furthermore, health care professionals in private hospitals are usually exposed to continuous professional education and training courses provided by their institutions to keep high standards of services and patient attraction [30], which could explain the study findings.

In addition, less than half of the participating healthcare providers reported the availability of AMS committees in their hospital settings, which limits the AMS implementation’s success. This challenge has been discussed by Mathew et al., as study participants confirmed the crucial need for an AMS committee or at least one AMS professional to refer to when needed [31]. Remarkably, most healthcare providers in the current study agreed that implementing AMS in the health institutions would improve the clinical health outcome; this is consistent with a study conducted by Alghamdi et al. in Saudi Arabia [32]. They reported that despite the low levels of AMS implementation in Saudi hospitals, they exhibited a solid intention to adopt them, wildly where participants are convinced of the benefits of AMS in enhancing patient safety and care [32].

Interestingly, most participants in the current study confirmed the importance of implementing AMS programs to reduce antimicrobial resistance. This finding is crucial for the future spread and application of this program in Jordanian hospitals. As well, such results were proven by a study conducted by Di Pentima et al., in which they found a significant reduction in the incidence of emergent antibiotic resistance, targeted/non-targeted antibiotic use, and improvement in the quality of care after the application of the AMS program [33]. Furthermore, another study by Ren-Zhang et al. (2020) demonstrated that the involvement of all healthcare professionals in the use of antimicrobials is the most critical key to the success of AMS programs [34]. In addition, measurement of awareness and cognition of AMS among healthcare professionals is required to guide essential, mandatory steps in AMS education [34].

Unfortunately, the level of AMS program implementation was insufficient in this study, despite the availability of AMS strategies in Jordanian hospitals, including the treatment guidelines, which are the most common strategy as reported by the healthcare providers. This finding is consistent with many published studies in the Gulf region, Europe, Asia, and Africa [28,35,36]. A lack of adequate implementation of AMS and the handling of its strategies were associated with increased antimicrobial-resistant infections in the past five years [28]. On the other hand, the proper execution of treatment guidelines in health institutions should be vital in managing antibiotic misuse. This could be related to the clarity and easiness of guidelines implementation [35].

The implementation of AMS programs in Jordan has faced many obstacles, such as a lack of information technology supports. This was reported as the main barrier to delivering functional and effective AMS programs, followed by the lack of funding. A similar study conducted in Ghana reported the ‘Failure to enforce laws’ as a primary barrier. Their study recommended overcoming such challenges through “*education and training for prescribers, improvement of labs for microbe-specific treatment, purchase of lab items for testing, antibiotic use checks/audits, employment of more staff to build the workforce and development of local policies.*” [18]. Furthermore, Baraka et al. stated that poor skills and knowledge are essential contributors to the misuse/overuse of antibiotics [36]. They also proposed the need for internal policy and treatment guidelines to guarantee the safe use of antimicrobials [36]. In the Jordanian context, these findings highlight the crucial need for an immediate action plan in developing the technological aspect, which saves time and effort in communicating information and finally reflects on the success of AMS program implementation [37]. On the other hand, the allocation of specialized grants to support the AMS program would result in positive clinical outcomes and reduce the expenses and consumption of antibiotics. Such findings have been reviewed by Cowman et al. to demonstrate that using technology to enhance AMS impacts the Acute Care Setting [38].

This study has some limitations that need to be highlighted. First, most of the respondents were from the capital of Jordan and a few from outside it, which could limit the generalizability of the study findings. Moreover, the study relied on healthcare providers’ self-assessment to implement AMS, which could overestimate the implementation at their hospital sites. Additionally, in this study, a convenience sample of healthcare providers (physicians, pharmacists, and nurses) working at several Jordanian hospitals) was recruited to participate. The participating healthcare providers from private hospitals are more prominent than those from the public hospitals. Finally, the proportion of respondents from each profession (physician, pharmacist, nurse) was 1:1:1, which may not reflect the actual ratio of professionals practicing in Jordanian hospitals. As a result, it might not be appropriate to generalize the finding among other hospitals.

## 5. Conclusions

In general, the study participants had a good level of awareness but reported the insufficient implementation of AMS programs. The main barrier to AMS implementation was the lack of information technology support. The result of this study may draw the attention of policymakers to the importance of AMS and the barriers to its’ implementation, which could positively reflect on the effective implementation of AMS at their institution. Moreover, the results of this research may help support the practice of AMS in Jordanian tertiary hospitals, highlighting the importance of following the most recent international AMS strategies and the continuous education/awareness programs for healthcare providers.

## Figures and Tables

**Table 1:** Socio-demographic characteristics of the study sample (*n* = 157).

Parameter	Total *n* (%)	Physicians *n* = 53	Pharmacists *n* = 51	Nurses *n* = 53
Age, years, median (IQR)	31.0 (10.0)	33.0 (12.0)	29.0 (10.0)	29.0 (7.0)
Gender, *n* (%)				
○ Males	63 (40.1)			
○ Females	94 (59.9)			
Years of experience, median (IQR)	5.0 (7.0)	7.0 (8.0)	7.0 (7.0)	4.0 (6.0)
Hospital classification, *n* (%)				
○ Public	53 (33.8)	26 (49.1)	11 (3.9)	16 (30.2)
○ Private	104 (66.2)	27 (50.9)	40 (96.1)	37 (69.8)
Hospital location, *n* (%)				
○ North of Jordan	11 (7.0)	5 (9.4)	2 (3.9)	4 (7.5)
○ Central of Jordan	141 (89.8)	44 (83.0)	49 (96.1)	48 (90.6)
○ South of Jordan	5 (3.2)	4 (7.5)	0 (0.0)	1 (1.9)

IQR: Interquartile range.

**Table 2:** The level of implementation of antimicrobial stewardship activities (*n* = 157).

Statements	Total *n* = 157	Private *n* = 104	Public *n* = 53	*p*-Value #
	Yes, *n* (%)			
**Organizational structure**				
Does your hospital have an antimicrobial stewardship committee?	69 (43.9)	51 (49.0)	18 (34.0)	0.072
Does your hospital have a policy that requires prescribers to document in the medical record the dose, duration, and indication for all antibiotic prescriptions?	118 (75.2)	81 (77.9)	37 (69.8)	0.268
Based on national guidelines and local susceptibility, does your hospital have a hospital-specific treatment recommendation (guideline)?	98 (62.4)	72 (69.2)	26 (49.1)	0.014 *
**Reporting**				
Does your hospital use antimicrobial resistance levels/surveillance reports?	109 (69.4)	79 (76.0)	30 (56.6)	0.013 *
Does your facility have software to record antimicrobial susceptibility results?	100 (63.7)	66 (63.5)	34 (64.2)	0.932
Does your facility have any antimicrobial use reports?	102 (65.0)	69 (66.3)	33 (62.3)	0.612
**Resources**				
Does your hospital provide access to literature or evidence-based medicine for medical staff while delivering care?	95 (60.5)	69 (66.3)	26 (49.1)	0.036 *
**Education**				
Does your stewardship program provide education to prescribers and other relevant staff on optimal prescribing, adverse reactions from antibiotics, and antibiotic resistance?	95 (60.5)	69 (66.3)	26 (49.1)	0.036 *

# Using Chi-squared test, * significant at 0.05 significance level.

**Table 3:** Assessment of healthcare providers’ perception towards the importance of antimicrobial stewardship programs (*n* = 157).

Statements	Agree/Strongly Agree	Neutral	Disagree/Strongly Disagree
Perceived Importance of Antimicrobial Stewardship	*n* (%)	*n* (%)	*n* (%)
Antimicrobial stewardship will improve patient’s clinical outcomes	134 (85.4)	8 (5.1)	15 (9.5)
Antimicrobial stewardship will reduce antimicrobial resistance	125 (79.6)	11 (7.0)	21 (13.4)
Antimicrobial stewardship improves the cost-effectiveness of health care sectors	121 (77.1)	11 (7.0)	25 (15.9)
Antimicrobial stewardship improves the collaboration between healthcare providers	106 (67.5)	19 (12.1)	32 (20.4)

**Table 4:** Assessment of healthcare providers’ perception of the barriers to delivering functional and effective antimicrobial stewardship programs (*n* = 157).

Statements	Agree/Strongly Agree	Neutral	Disagree/Strongly Disagree
Perceived Barriers to Implementing Antimicrobial Stewardship	*n* (%)	*n* (%)	*n* (%)
Lack of sufficient healthcare providers	119 (75.8)	12 (7.9)	26 (16.6)
Lack of funding	121 (77.1)	13 (8.3)	23 (14.6)
The hospital administration is not aware of antimicrobial stewardship program	103 (65.6)	20 (12.7)	34 (21.7)
The antimicrobial Prescribers are not aware of antimicrobial stewardship program	110 (70.1)	17 (10.8)	30 (19.1)
Opposition from prescribers	116 (73.9)	19 (12.1)	22 (14.0)
Lack of information technology support	125 (79.6)	11 (7.0)	21 (13.4)
Lack of resources to get the needed data	118 (75.2)	11 (7.0)	28 (17.8)

## Data Availability

Data is contained within the article or Supplementary Material.

## Supplementary Materials

The following supporting information can be downloaded at: https://www.mdpi.com/article/10.3390/antibiotics11010099/s1, File S1: Study Questionnaire.
