# Supplementary material for: Antimicrobial Stewardship from Health Professionals’ Perspective: Awareness, Barriers, and Level of Implementation of the Program
Source: Antibiotics (Basel). 2022 Jan 14;11(1):99. doi: 10.3390/antibiotics11010099 (PMC8773352; doi:10.3390/antibiotics11010099)
Supplement: Supplementary file 1 [file antibiotics-11-00099-s001.zip › antibiotics-1522325-supplementary.pdf]

Study Questionnaire

Antimicrobial Stewardship from health professionals' perspective: Awareness, barriers, and level of implementation of the program

Informed Consent Form

Dear participant

Researchers from Applied Science Private University are carrying out a research project with the purpose of evaluating the extent of the antimicrobial stewardship practice in Jordanian tertiary hospitals from healthcare providers' (physicians, pharmacists, and nurses) perspective.

For confidentiality, the surveys will not include information identifying your personal identity and will be used only for research purposes.

The procedure involves filling an online survey that will take approximately five minutes. Your participation is highly appreciated.

ELECTRONIC CONSENT: Please select your choice below.

Clicking on the "agree" button below indicates that:

1. You have read the above information
2. You voluntarily agree to participate

If you do not wish to participate in the research study, please decline participation by clicking on the "disagree" button.

- ❑ Agree
- ❑ Disagree

Part 1: Demographic characteristics of healthcare providers

Age: ________________ (years)

Gender:

- ❑ Male
- ❑ Female

Healthcare discipline
- ❑ Physician
- ❑ Pharmacist
- ❑ Nurse

Experience in your current specialty or profession: ______________________________

Hospital facility classification
- ❑ Public
- ❑ Private

Name of the hospital: ___________________

Hospital facility location
- ❑ North region (Irbid, Ajloun, Jerash, Mafraq)
- ❑ Central region (Amman, Zarqa, Balqa, Madaba)
- ❑ South region (Karak, Tafilah, Ma`an, Aqaba)

Are you aware about the presence of the National Action Plan on antimicrobial stewardship (2018–2022) in Jordan?
- ❑ Yes
- ❑ No

Assessment of antimicrobial stewardship practice in hospital settings

Definition
Antimicrobial stewardship refers to a coordinated intervention designed to improve the appropriate use of antibiotics by promoting the selection of optimal antimicrobial drug regimen, dose, duration of therapy and route of administration

Organizational structure

Does your hospital have an antimicrobial stewardship committee?
- ❑ Yes
- ❑ No
- ❑ Not sure

Does your hospital have a policy that requires prescribers to document in the medical record of during entry a dose, duration, and indication for all antibiotic prescriptions?
- ❑ Yes

- ❑ No
- ❑ Not sure

Based on national guidelines and local susceptibility, does your hospital have a hospital-specific treatment recommendation (guideline)?

- ❑ Yes
- ❑ No
- ❑ Not sure

Please choose the available antibiotic stewardship strategy in your hospitals (Multiple answers is possible)

- ❑ Treatment guidelines
- ❑ Surgical prophylaxis guidelines
- ❑ Antimicrobial cycling
- ❑ Prospective audit feedback
- ❑ Antimicrobial formulary
- ❑ Intravenous-to-oral switch guidance
- ❑ Empiric usage form
- ❑ Restricted antimicrobial list
- ❑ Streamlining or de-escalation of therapy
- ❑ Pre-authorized pharmacy-driven dose optimization (e.g., automatic renal dose adjustments, intravenous-to-oral conversions, etc.)
- ❑ Infectious diseases/microbiology advice by telephone
- ❑ Infectious diseases/microbiology advice on ward rounds
- ❑ Separate antimicrobial chart or section

Does your hospital use antimicrobial resistance levels/surveillance reports?

- ❑ Yes
- ❑ No
- ❑ Not sure

Does your facility have software to record antimicrobial susceptibility results?

- ❑ Yes
- ❑ No
- ❑ Not sure

Does your facility have any antimicrobial use reports?

- ❑ Yes
- ❑ No
- ❑ Not sure

Does your hospital provide access to literature or evidence-based medicine for medical staff while delivering care?

- ❑ Yes

- ❑ No
- ❑ Not sure

Does your stewardship program provide education to prescribers and other relevant staff on optimal prescribing, adverse reactions from antibiotics, and antibiotic resistance?

- ❑ Yes
- ❑ No
- ❑ Not sure

Perceived importance of antimicrobial stewardship

Please choose the level of agreement with the following statements, reflecting the importance of antimicrobial stewardship implementation in the hospital setting.

**Statements**	Strongly agree	Agree	Neutral	Disagree	Strongly disagree
Antimicrobial stewardship will improve patient's clinical outcomes	❑	❑	❑	❑	❑
Antimicrobial stewardship will reduce antimicrobial resistance	❑	❑	❑	❑	❑
Antimicrobial stewardship improves the cost-effectiveness of health care sectors	❑	❑	❑	❑	❑
Antimicrobial stewardship improves the collaboration between healthcare providers	❑	❑	❑	❑	❑

Barriers to delivering a functional and effective antimicrobial stewardship

Please choose the level of agreement with the following statements, reflecting the barriers of antimicrobial stewardship implementation in the hospital setting.

Statements	Strongly agree	Agree	Neutral	Disagree	Strongly disagree
Lack of sufficient healthcare providers	❑	❑	❑	❑	❑
Lack of funding	❑	❑	❑	❑	❑
The hospital administration is not aware of AMS program	❑	❑	❑	❑	❑
The antimicrobial Prescribers are not aware of AMS program	❑	❑	❑	❑	❑
Opposition from prescribers	❑	❑	❑	❑	❑
Lack of information technology support	❑	❑	❑	❑	❑
Lack of resources to get the needed data	❑	❑	❑	❑	❑
